# Supplementary material for: Effects of childhood and adult height on later life cardiovascular disease risk estimated through Mendelian randomization
Source: Eur J Epidemiol. 2025 Mar 19;40(2):167–76. doi: 10.1007/s10654-025-01203-2 (PMC12018521; doi:10.1007/s10654-025-01203-2)
Supplement: Supplementary file 1 — Supplementary Material 1 [file 10654_2025_1203_MOESM1_ESM.docx]

**Supplementary Note 1**

#### Genetic correlation analyses

Genetic correlations of our GWAS results with measured childhood and adult height from independent samples supported their utility as genetic instruments capable of separating effects at these timepoints. Genetic estimates identified in our childhood GWAS had a higher correlation with measured childhood height compared to our adult GWAS findings in UKB (rG=0.90 (95% CI=0.82-0.98) vs rG=0.75 (95% CI=0.67-0.83) respectively). In contrast, our adult height estimates were very strongly correlated with measured adult height in an independent sample (rG=0.97 (95% CI=0.96-0.99)) compared to results from our childhood height GWAS (rG=0.87 (95% CI=0.85-0.89)).

#### The Avon Longitudinal Study of Parents and Children

The Avon Longitudinal Study of Parents and Children (ALSPAC) is a population-based cohort study investigating genetic and environmental factors that affect the health and development of children. The study methods are described in detail elsewhere(Boyd et al., 2019). Briefly, 14 541 pregnant women residing in the former region of Avon, UK, with an expected delivery date between 1 April 1991 and 31 December 1992 were eligible to take part in ALSPAC. Detailed phenotypic information, biological samples, and genetic data have been collected from the participants, which are available through a searchable data dictionary (<http://www.bristol.ac.uk/alspac/researchers/our-data/>). In this study, we used data on clinically measured height to the nearest cm to validate genetic instruments identified in UKB. Data on height in ALSPAC was analysed at two timepoints over the life course. These were at the age 9 offspring clinic (mean age: 9.9 years) and the focus on mothers 1 clinic (mean age: 47.9 years). Written informed consent was obtained for all study participants. Ethical approval for the study was obtained from the ALSPAC ethics and law committee and the local research ethics committees.

Using data from ALSPAC we firstly constructed genetic risk scores using data from the ALSPAC study and evaluated their association with clinically measured height during childhood (mean age: 9.9 years) and adulthood (mean age: 47.9 years). Height variables were dichotomised into halves to derive binary variables indicating whether individuals were above or below average height at each timepoint. Receiver operator characteristic (ROC) curves were generated to evaluate each set of genetic instruments’ capability to predict measured height at both timepoints in ALSPAC. Additionally, we undertook linkage disequilibrium score regression to estimate the genetic correlation between the two measures of height we derived in UKB and GWAS of measured childhood and adult height in two non-overlapping samples (Bulik-Sullivan et al., 2015).

#### The 1958 National Child Development Study

The 1958NCDS is a longitudinal assessment of 17,415 individuals who were born within a single week in March 1958 (Power and Elliott, 2006). Beginning at the week of birth, mothers and their children were repeatedly assessed at irregular intervals, using a comprehensive set of measurements and assessments regarding many aspects of their lives. We analysed a subset of 5847 individuals who had undergone genome-wide array-based genotyping and imputation, and who were inferred to be of European descent (again using genetic principal component analysis). Of these 5847 individuals, 3190 had measures of height (in metres) at age 7, 11 and 16 years, and 2857 measures ages 23 and 44. Data at ages 33 and 50 were also recorded as part of the study, but age 33 was excluded due to extreme values. Height at age 50 was excluded due to a lack of coverage (N=47).

We determined the squared residual explained by the two genetic scores by linear regression of the genetic score on height residualised on sex and geographical location. Both the genetic risk score and height were also transformed to z-scores. We additionally determined the AUC for each time-point by contrasting the predictive score with a binary measure for whether an individual’s height was 1SD higher than the population mean.

**Supplementary Figure 1**


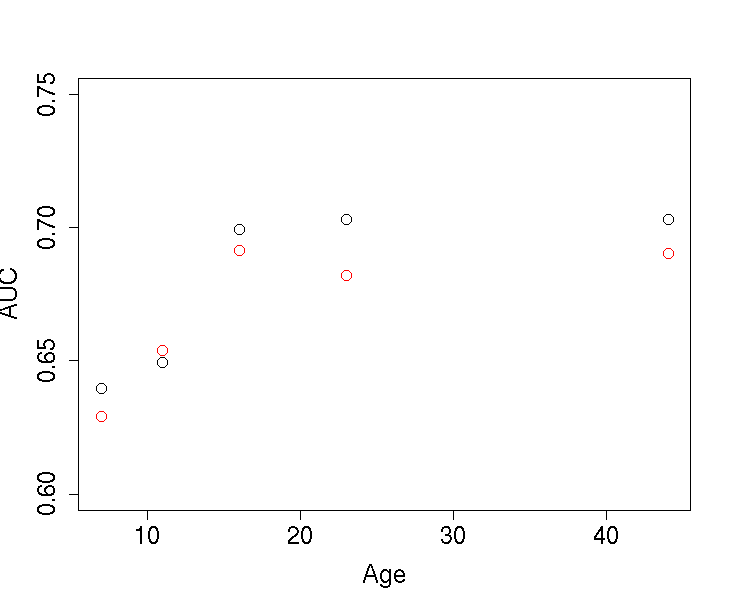


#### Childhood and adult height variance explained (r^2^) as a function of time (childhood score results are shown by red circles, adulthood by black)

**Supplementary Figure 2**


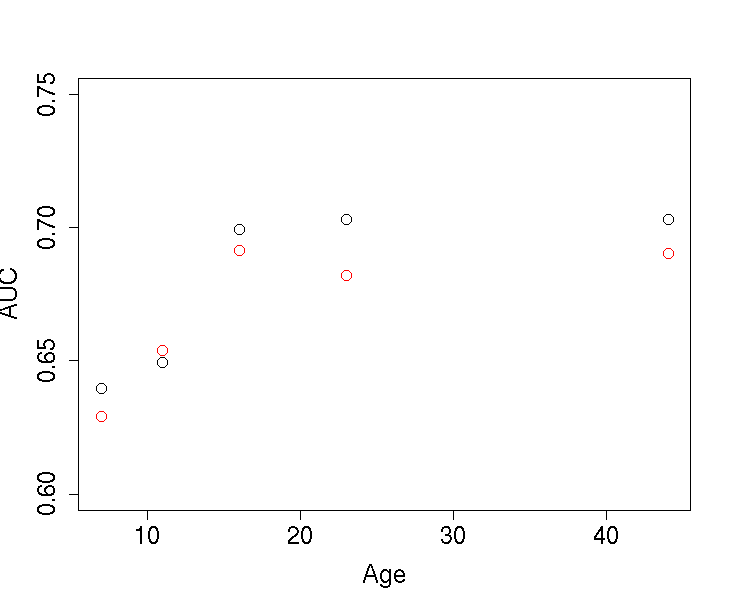


#### Childhood and adult height area under curve (AUC) as a function of time (childhood score results are shown by red circles, adulthood by black)

#### Validation of genetic instruments using within-family models

We attempted to validate the genetic instruments for childhood height using within-sibiship models which are robust against effects of population stratification, assortative mating and dynastic effects (Brumpton et al., 2020). As above, we used PLINK to LD clump the UK Biobank GWAS data (which excluded the siblings) and then constructed adult height/childhood height genetic risk scores in the sample of 38,807 siblings. We then fit within-sibship and population regression models (below) to estimate the association between the genetic risk scores and phenotypes including sex, birth year and the first principal components as covariates. The within-sibship model included the mean genotype of siblings in each family as an additional covariate to account for family structure. Standard errors from both estimators were clustered over sibships to account for non-random clustering of siblings within families (Lee et al., 2018).

For individual $j$ in sibship $i$ with $n_{i}\geq2$ siblings:

Population model:

$${Phen}_{ij} \sim G_{ij}+{Sex}_{ij}+{Age}_{ij}+{PC1}_{ij}+.. {PC20}_{ij}$$

Within-sibship model:

$${Phen}_{ij} \sim G_{ij}^{C}+F_{i}+ {Sex}_{ij}+{Age}_{ij}+{PC1}_{ij}+.. {PC20}_{ij}$$

where $F_{i}= \frac{G_{i1}+..G_{in}}{n_{i}}$ and $G_{ij}^{C}= G_{ij}-F_{i}$

To compare the PGS associations under the population and within-sibship models, we estimated the within-sibship shrinkage (WSS), i.e. the attenuation in the PGS-phenotype association from the population to within-sibship models. Standard errors were estimated using leave-one-out jackknife resampling. This involved removing each family k (of 18,852) and estimating the standard error as follows where WSS_k_ is the shrinkage estimate from the sample with sibship k removed:

$$WSS = 1- \frac{\beta_{WF}}{\beta_{Conventional}}$$

$$SE\left( WFS \right)=\sqrt{\frac{18851}{18852}\sum_{1}^{18852} ({WFS}_{k}-\mu})^{2}$$

where $\mu=\frac{\sum_{1}^{18852} {WFS}_{k}}{18852}$

Sibling paragraph from Laurence

|  | Population association:  Unit increase per SD increase in PGS (95% C.I.) | Within-sibship association:  Unit increase per SD increase in PGS (95% C.I.) | Within-sibship shrinkage:  % (95% C.I.) |
| --- | --- | --- | --- |
| Childhood height GRS and childhood height  (add info on 0,1,2 scale) | 0.21 (0.20, 0.22) | 0.21 (0.20, 0.22) | -2% (-7%, 3%) |
| Adult height GRS and adult height  (add info on 0,1,2 scale) | 0.20 (0.19, 0.20) | 0.18 (0.17, 0.18) | 10% (6%, 14%) |

Boyd, A., Thomas, R., Hansell, A. L., Gulliver, J., Hicks, L. M., Griggs, R., Vande Hey, J., Taylor, C. M., Morris, T., Golding, J., Doerner, R., Fecht, D., Henderson, J., Lawlor, D. A., Timpson, N. J. and Macleod, J. (2019) 'Data Resource Profile: The ALSPAC birth cohort as a platform to study the relationship of environment and health and social factors', *Int J Epidemiol,* 48(4), pp. 1038-1039k.

Brumpton, B., Sanderson, E., Heilbron, K., Hartwig, F. P., Harrison, S., Vie, G. A., Cho, Y., Howe, L. D., Hughes, A., Boomsma, D. I., Havdahl, A., Hopper, J., Neale, M., Nivard, M. G., Pedersen, N. L., Reynolds, C. A., Tucker-Drob, E. M., Grotzinger, A., Howe, L., Morris, T., Li, S., Within-family, C., andMe Research, T., Auton, A., Windmeijer, F., Chen, W. M., Bjorngaard, J. H., Hveem, K., Willer, C., Evans, D. M., Kaprio, J., Davey Smith, G., Asvold, B. O., Hemani, G. and Davies, N. M. (2020) 'Avoiding dynastic, assortative mating, and population stratification biases in Mendelian randomization through within-family analyses', *Nat Commun,* 11(1), pp. 3519.

Bulik-Sullivan, B. K., Loh, P. R., Finucane, H. K., Ripke, S., Yang, J., Schizophrenia Working Group of the Psychiatric Genomics, C., Patterson, N., Daly, M. J., Price, A. L. and Neale, B. M. (2015) 'LD Score regression distinguishes confounding from polygenicity in genome-wide association studies', *Nat Genet,* 47(3), pp. 291-5.

Lee, J. J., Wedow, R., Okbay, A., Kong, E., Maghzian, O., Zacher, M., Nguyen-Viet, T. A., Bowers, P., Sidorenko, J., Karlsson Linner, R., Fontana, M. A., Kundu, T., Lee, C., Li, H., Li, R., Royer, R., Timshel, P. N., Walters, R. K., Willoughby, E. A., Yengo, L., andMe Research, T., Cogent, Social Science Genetic Association, C., Alver, M., Bao, Y., Clark, D. W., Day, F. R., Furlotte, N. A., Joshi, P. K., Kemper, K. E., Kleinman, A., Langenberg, C., Magi, R., Trampush, J. W., Verma, S. S., Wu, Y., Lam, M., Zhao, J. H., Zheng, Z., Boardman, J. D., Campbell, H., Freese, J., Harris, K. M., Hayward, C., Herd, P., Kumari, M., Lencz, T., Luan, J., Malhotra, A. K., Metspalu, A., Milani, L., Ong, K. K., Perry, J. R. B., Porteous, D. J., Ritchie, M. D., Smart, M. C., Smith, B. H., Tung, J. Y., Wareham, N. J., Wilson, J. F., Beauchamp, J. P., Conley, D. C., Esko, T., Lehrer, S. F., Magnusson, P. K. E., Oskarsson, S., Pers, T. H., Robinson, M. R., Thom, K., Watson, C., Chabris, C. F., Meyer, M. N., Laibson, D. I., Yang, J., Johannesson, M., Koellinger, P. D., Turley, P., Visscher, P. M., Benjamin, D. J. and Cesarini, D. (2018) 'Gene discovery and polygenic prediction from a genome-wide association study of educational attainment in 1.1 million individuals', *Nat Genet,* 50(8), pp. 1112-1121.

Power, C. and Elliott, J. (2006) 'Cohort profile: 1958 British birth cohort (National Child Development Study)', *Int J Epidemiol,* 35(1), pp. 34-41.
